# Redox Biology of Infection and Consequent Disease

**DOI:** 10.1155/2020/5829521

**Published:** 2020-01-27

**Authors:** Maria G. Isaguliants, Birke Bartosch, Alexander V. Ivanov

**Affiliations:** ^1^Riga Stradins University, LV-1007 Riga, Latvia; ^2^Department of Microbiology, Tumor and Cell Biology, Karolinska Institutet, 17177 Stockholm, Sweden; ^3^N.F. Gamaleja Research Center of Epidemiology and Microbiology, 123098 Moscow, Russia; ^4^Chumakov Federal Scientific Center for Research and Development of Immune-and-Biological Products of Russian Academy of Sciences, 108819 Moscow, Russia; ^5^Cancer Research Center Lyon, INSERM U1052 and CNRS 5286, Lyon University, 69003 Lyon, France; ^6^DevWeCan Laboratories of Excellence Network (Labex), France; ^7^Center for Precision Genome Editing and Genetic Technologies for Biomedicine, Engelhardt Institute of Molecular Biology, Vavilov str, 32, 119991 Moscow, Russia

Bacteria, viruses, and parasites are the etiological agents that trigger various diseases referred to as communicable. According to WHO statistics, they are responsible for 16% of deaths worldwide. Many of these agents cause severe morbidities already during the initial infection but can also persist in their host and cause long-term inflammation; fibrosis; and cardiovascular, neurodegenerative, and autoimmune diseases. Extensive research over the last two decades demonstrated that many of these chronic infections are associated with elevated production of reactive oxygen species (ROS) and reactive nitrogen species (RNS). Enhanced ROS/RNS production has been implicated in numerous pathologies such as fibrosis, cirrhosis, metabolic dysfunction, lung tissue injury, and epithelial barrier dysfunction, which in turn often increase the susceptibility to secondary infections and/or trigger cancer. Redox biology significantly advanced our understanding of these pathologies. Furthermore, tremendous progress has been achieved in understanding how ROS/RNS affect signaling pathways. However, still too little is known about the mechanisms by which pathogens manipulate oxidative/nitrosative stress reaction(s) of the host. With this in mind, we invited original research and review articles on redox biology of infections and associated pathologies, as well as studies that mechanistically explore redox biology that in the future may be applicable to infection studies.

Infectious agents induce oxidative/nitrosative stress by different mechanisms. Amongst the host or pathogen enzymes which produce ROS/RNS and are induced or activated in the process of infection, particular attention in the past was drawn to the electron transport chain of mitochondria and NADPH oxidases. Other ROS-producing systems, mentioned in our review on hepatitis B and C viruses [1], remained largely ignored. To fill this gap, a comprehensive review of A. V. Snezhkina et al. presents a thorough summary of the activities of a (broad) panel of ROS-generating enzymes and pathways. They also discuss influence of glycolysis and adjacent metabolic pathways on the redox status of cells, as they affect NADPH/NADP and NADH/NAD ratio and, thus, may change the overall redox balance.

Extensive data have accumulated on the redox biology of viral infections, including viral hepatitis B and C, human immunodeficiency virus (HIV-1), influenza viruses, herpes-, respiratory syncytial, rhino-, corona-, and papillomaviruses. Particularly well studied is the redox biology of hepatitis C virus. During last two decades, numerous papers reported how HCV triggers oxidative stress, how it affects the antioxidant defense systems of the host cell, and what virus-associated pathologies develop due to continuous production of ROS. As reviewed by Z. Zhang et al., most of the HCV proteins are capable of inducing oxidative stress, including core, E1, E2, NS3, NS4B, and NS5A. Of these, core stands as a key inducer of calcium perturbations and ROS production, which in turn regulate the viral replication cycle by molecular mechanisms that remain largely unknown. M. K. Kukhanova et al. present exciting novel concepts to fill this gap. They have demonstrated that the RNA-dependent RNA polymerase encoded by HCV undergoes S-glutathionylation and that this modification affects enzymatic activity of the protein. Thus, M. K. Kukhanova et al. showed that this protein is a bona fide “redox switch” with activity/functions regulated through redox-dependent posttranslational modifications of cysteine residues (such as S-glutathionylation, nitration, and oxidation to sulfenic, sulfinic, and sulfonic acids) [2].

Zika and dengue viruses belong to the same family as HCV, the Flaviviridae. The redox biology of these pathogens is much less studied, despite association of these viruses with severe human diseases. In a comprehensive review, Z. Zhang et al. carefully discuss the current state of the art for different members of *Flaviviridae* family and for the whole Flavivirus genus. Many of these viruses cause cell damage by generating ROS and changing redox homeostasis. In turn, ROS facilitate viral replication in variable ways, depending on the cell type and involved virus, but in general, both flaviviruses and alphaviruses use oxidative stress produced during infection to temporally control genome RNA capping and genome replication. According to Z. Zhang et al., viruses vary in their ability to induce ROS but share a common pathway to defend the infected cell against oxidative stress which involves nuclear factor erythroid 2 p45-related factor 2 (Nrf2), a master transcriptional factor regulating a panel of antioxidant and cellular defense genes in response to oxidative stress.

Hepatitis B virus (HBV) of *Hepadnaviridae* family is another pathogen which, similar to HCV, causes liver disease characterized by chronic liver inflammation, fibrosis, cirrhosis, and eventually liver cancer. Noteworthily, there are currently no approved drugs, which can cure this infection. Many attempts were made to unveil the impact of HBV on the redox status of infected hepatocytes. Not surprisingly, several independent groups revealed that overexpression of single individual HBV proteins promotes production of ROS and pointed to mitochondria and endoplasmic reticulum as their most likely source. HBV core and surface antigens (HBc and HBs) were claimed responsible for induction of ER stress as well as the event(s) occurring during accumulation of the misfolded proteins, a process closely linked to the production of ROS. No specific mechanisms for these events have yet been proposed. In this issue, M. Mehmankhah et al., albeit not studying this process directly, presented a model of HBs structure and, based on this model, performed an *in silico* search for small molecular weight ligands that block HBV infection. This research on HBs folding will lead to the elucidation of mechanisms behind the accumulation of misfolded HBs and resultant oxidative stress. As the efflux of Ca^2+^ ions from ER to mitochondria and mitochondrial dysfunction are the direct consequences of ER stress, this study will aid our understanding of the molecular/structural basis of HBV's influence on ROS production and calcium homeostasis.

Main bulk of data in the viral redox biology concerns human pathogens while the knowledge on their animal counterparts remains obscure. In this special issue, X. Fu et al. studied the status of the Nrf2/ARE pathway in cells infected with Bovine herpesvirus type 1 (BoHV-1) that is a significant cofactor for bovine respiratory disease complex, the most important inflammatory disease in cattle. They demonstrated that BoHV-1 infection in cell culture induces overproduction of ROS and depletion of Nrf2 and its downstream targets heme oxygenase-1 and NAD(P)H quinone oxidoreductase-1 proteins. BoHV-1 inhibited the Nrf2 signaling pathway by complicated mechanisms including promoting Nrf2 degradation, relocalization of nuclear Nrf2, and inhibition of Nrf2 acetylation. This may account for or at least contribute to the pathogenicity of this infection in cattle.

Tissue inflammation and regeneration processes induced in multiple chronic microbial infections evolve over long periods of time in the context of a strongly oxidative microenvironment leading to mutations in many cellular signaling cascades that drive cell growth and proliferation. This makes oxidative stress a driving force of the infection-induced or infection-associated neoplastic transformation [1]. The best studied oncogenic viruses are human papillomaviruses (HPV), associated with anal, cervical, oropharyngeal, and penile cancer; hepatitis B and C viruses (HBV and HCV), associated with liver cancer; and Epstein-Barr virus, associated with Burkitt's lymphoma, Hodgkin's disease, and nasopharyngeal carcinoma. The oncogenic potential of HIV has for a long time been thought to be due to the virus-mediated immune suppression. However, HIV-infected people were also found to be at an increased risk of “non-AIDS-defining” malignancies not directly linked to immune suppression but associated with other viral infections. Their incidence has been increasing despite successful antiretroviral therapy. The mechanism behind this phenomenon is unclear. E. Bayurova et al. obtained daughter clones of murine mammary gland adenocarcinoma 4T1luc2 cells expressing variants of consensus HIV-1 reverse transcriptase (RT) and found that expression of these RT variants in 4T1luc2 cells leads to increased production of ROS, lipid peroxidation, enhanced cell motility in the wound healing assay, and upregulation of expression of Vimentin and Twist. Importantly, when implanted into syngeneic BALB/C mice, 4T1luc2 cells expressing HIV-1 RT demonstrated enhanced rate of tumor growth and increased metastatic activity. This study establishes links between the expression of HIV-1 RT, production of ROS, induction of EMT, and enhanced propagation of RT-expressing tumor cells. E. Bayurova et al. proposed this scenario as one of the mechanisms of HIV-induced/enhanced carcinogenesis not associated with immune suppression. This study reinforces the earlier findings on the mechanisms of HIV-1-induced production of ROS and effect of ROS on HIV-1 replication [3].

In their review, A. V. Snezhkina et al. described the molecular basis underlying carcinogenesis. They have listed positive effects of ROS, including activation of apoptotic pathways and aiding anticancer treatments, but also described ROS-mediated mechanisms of carcinogenesis, including DNA damage, leading to the accumulation of mutations and genome instability, as well as reprogramming of cell metabolism and signaling. Which properties of HIV-1 RT are responsible for the induction of ROS, if not the enzymatic activities, remained unknown. E. Bayurova et al. refer to a review of cellular interactions of HIV-1 RT, listing factors which directly or indirectly bind to RT and regulate reverse transcription but at the same time are involved in the maintenance of the redox balance of the cell, the latter possibly affected by their interaction with RT [4].

Bacterial infections traditionally have not been considered to be a major cause of cancer. Recently, however, it has been reported that microbiota can promote carcinogenesis by inducing oxidative stress, genotoxicity, host immune response disturbance, and chronic inflammation. A vivid example is carcinogenesis induced by infection with *Helicobacter pylori* infection [4]. There is also increasing data on the role for oxidative stress in salmonellosis, tuberculosis, and pseudomonas infections. In this context, the search for bioactive substances capable to modulate excessive ROS production in the host represents a new direction in the development of new antimicrobial and, potentially, anticancer drugs. Schiff bases (SBs) are chemical compounds displaying a significant pharmacological potential. In this issue, C. C. Login et al. presented a new thiazolyl-triazole SB with antibacterial activity against *L. monocytogenes* and *P. aeruginosa*, being two times more active than ciprofloxacin. Antibacterial activity on the Gram-negative bacilli was attributed to the decrease of the bacterial NO synthase level and formation of complexes with metals located in the active center of certain bacterial enzymes. Importantly, this new compound showed radical scavenging activity, attributable to the presence of –SH group. The effect of SB on oxidative stress tested in cell culture revealed a decrease in the lipid peroxidation and the protein level of enzymes COX2 and an iNOS involved in the modulation of oxidative stress and inflammatory response.

Positive effects of antioxidants were also recorded in another study presented in this issue by E. K. Fetisova et al., who assessed the therapeutic effect of the mitochondria-targeted antioxidant SkQ1 on a culture model of Multiple Sclerosis (MS). MS is a heterogeneous autoimmune disease of unknown etiology characterized by inflammation, demyelination, and axonal degeneration affecting both the white and grey matter of the CNS. Events leading to the activation of immune cells in MS are mostly unknown; however, damage to the nerve tissue by MS lesions resembles those observed in infectious leprosy [5]. The main cause of the nerve damage was suggested to be nitrozooxidative stress [6]. With this in mind, E. K. Fetisova et al. studied the effect of mitochondria-targeted antioxidant SkQ1 in an *in vitro* model of MS, primary oligodendrocyte culture of the cerebellum, challenged with lipopolysaccharide (LPS). SkQ1 accumulated in the mitochondria of microglial cells and in oligodendrocytes, restoring their capacity to produce myelin, initially blocked by LPS. These results implicate that mitochondria-targeted antioxidants could be promising components of a combined therapy for MS and related neurological disorders, including bacterial infections of the nervous tissues, as an alternative therapy for the cases where immunomodulating disease-modifying therapy is not available or not possible. The latter relates to immunosuppressed individuals, who are at an increased risk of treatment complications due to polyoma- and herpesvirus infections [7]. In contrast to immunomodulatory drugs, mitochondria-targeted antioxidants do not cause any signs of immunosuppression and would not cause such complications.

Emerging evidence indicates that certain parasites such as the blood fluke *Schistosoma haematobium* and small liver flukes *Opisthorchis viverrini* and *Clonorchis sinensis* can also serve as causative agents of malignancies such as bladder cancer caused by schistosomes and cholangiocarcinoma by liver flukes. Recent studies revealed that the generation of ROS and RNS associated with oxidative stress plays a crucial role in the development of systemic complications caused by malaria [8]. In this journal issue, D. M. S. Pereira et al. revealed that another manifestation of *P. falciparum* pathogenicity associated with oxidative stress, namely, cerebral malaria, is mediated through the ROS-dependent functions of housekeeping transient receptor potential (TRP) channels. Cerebral malaria is a clinical syndrome of a severe form of the disease characterized by neurological complications (coma and convulsions) associated with brain inflammation [9]. Several mechanisms were found to contribute to cerebral malaria including alterations in nitric oxide availability, unbalanced oxidative stress responses, and brain inflammation [10]. TRP channels are nonselective cationic channels, distributed in a diverse range of tissues, with local expression in the free terminals of nociceptive nerve fibers and skin [11], involved in the direct detection of stimuli associated with senses and maintenance of ionic homeostasis [12]. TRPV1 plays an important role in the physiology of the digestive system, cardiovascular system, and respiratory system, as well as the development of various pathologies through evoking an inflammatory response [13, 14]. D. M. S. Pereira et al. for the first time demonstrated that mice infected with *P. berghei* exhibited lower levels of expression of TRPV1 than noninfected controls. In the absence of TRPV1 expression, infection with *P. berghei* did not progress to cerebral malaria. The majority of the infected mice remained protected against the development of any disease symptoms and signals apart from blood parasitaemia. Similar protection was provided by treatment with the TRPV1 antagonist SB366791. This study underpinned the molecular mechanisms linking parasite infection with regulation of production of ROS [15] by the host, each and both affecting host cell machinery.

TRPV1 is linked with both the process of inflammation and of calcium signaling and thus may contribute to cancer progression [16]. This creates an association between a decrease in expression of TRPV1 as a factor of selection predisposing to survival from the cerebral malaria and at the same time increasing risk(s) of acquisition of cancer. A similar association was noted for Duffy Antigen Receptor for Chemokines expressed by red blood cells [17]. Whether these “malaria-cancer” associations actually translate into increased risks of induction cancer, specifically of Burkitt lymphomas, in individuals infected with malaria [18] remains to be elucidated. Even with this inclarity, the main message of this special issue remains: oxidative stress caused by infections, especially chronic infections, directly or indirectly potentiates neoplastic transformation.
